# Assessment of adverse events related to anti-influenza neuraminidase inhibitors using the FDA adverse event reporting system and online patient reviews

**DOI:** 10.1038/s41598-020-60068-5

**Published:** 2020-02-20

**Authors:** Nayoung Han, Jung Mi Oh, In-Wha Kim

**Affiliations:** College of Pharmacy and Research Institute of Pharmaceutical Sciences, Seoul National University, Seoul, Republic of Korea

**Keywords:** Medical research, Risk factors

## Abstract

The recommended antiviral drugs available for the treatment and prevention of influenza are neuraminidase inhibitors (NAIs). The aim of this study was to evaluate age-related clinical manifestations of adverse events (AEs) related to NAIs. FAERS and WebMD data were downloaded. The available NAIs selected for the analysis were oseltamivir, peramivir, zanamivir, and laninamivir. Disproportionality was analyzed using the proportional reporting ratio (PRR), the reporting odds ratio (ROR), and the information component (IC) methods. In total, 16729 AEs from 4598 patients and 575 AEs from 440 patients in the FAERS and WebMD, respectively, were included in the analysis. In the FAERS, AEs were more common among those who were younger (<19 years) for zanamivir, while for those who were older (>65 years) for peramivir. A disproportionality analysis showed that signals for vomiting and hallucinations were detected in younger patients given oseltamivir, while an abnormal hepatic function, cardiac failure, shock, and cardio-respiratory arrest were detected in older patients given peramivir. Psychiatric disorders were most common in younger and older patients, while gastrointestinal disorders were most common in adult given oseltamivir in the WebMD. Adverse symptoms related to NAIs varied and depended on the drugs used and the age of the patient.

## Introduction

Influenza remains a major threat to public health in spite of the fact that an influenza vaccination has been shown to be effective for preventing infection. Every year worldwide, there are an estimated one billion cases of influenza, of which approximately three to five million are severe cases, resulting in 290,000 to 650,000 influenza-related deaths^1^. The recommended antiviral drugs available for the treatment and prevention of influenza are neuraminidase inhibitors (NAIs)^2^. After oseltamivir and zanamivir were approved by the U.S. Food and Drug Administration (FDA) in 1999, peramivir and laninamivir were developed for the prevention and control of influenza^3,4^, peramivir and laninamivir were developed for the prevention and control of influenza^5,6^. The two NAIs oseltamivir and zanamivir were widely used during the 2009 influenza A (H1N1) pandemic^7^. The most common adverse effects for oseltamivir are nausea, vomiting, and diarrhea^8^. Zanamivir can cause bronchospasm when administered by inhalation^4^. The U.S. FDA has recently approved the intravenous use of peramivir^5^, but has not approved laninamivir for use.

Reports mostly from Japan in 2005 and 2006 suggested that oseltamivir increased the risk of neuropsychiatric symptoms such as delirium, hallucinations, and abnormal behaviors, and could lead to thoughts of self-injury or suicide in teenagers^9^. However, the U.S. FDA’s pediatrics advisory committee concluded that the deaths were not related to oseltamivir, though twelve deaths were reported in Japanese children who were taking it in 2005^10^. In 2006, therefore, the U.S. FDA added a warning to the label of oseltamivir, drawing attention to the risk of developing neuropsychiatric adverse events (AEs) such as confusion and abnormal behaviors^11^.

In 2007, there were reports of approximately 100 cases and 70 deaths of abnormal behaviors associated with oseltamivir among children and adolescents in Japan^12^. There have also been several case reports of neuropsychiatric events associated with oseltamivir in countries other than Japan^13–16^. These side effects have been reported in children, adolescents, and young adults in particular^17,18^.

Recently, the Drug Safety Subcommittee of Ministry of Health, Labour and Welfare, Japan (MHLW) reviewed the warning texts of anti-influenza drugs for revisions based on the study results over the last 10 years and concluded that attention should be paid to the risk of abnormal behaviors in all patients with influenza, irrespective of treatment with NAIs. Additionally, the warnings for the abnormal behaviors should be given to the patients less than 20 years old, regardless of ages^9^.

On the other hands, senior patients bear the greatest burden with 50% to 70% of seasonal influenza-related hospitalizations and 70% to 90% seasonal influenza-related deaths^19^. Yet, it is not well known about NAIs related AEs in these patients.

Suspected adverse drug events were voluntary reported to spontaneous reporting systems, and this system database was used to search for signals between drugs and adverse effects^20,21^. Additionally, internet message boards provide evidence of the increasing frequency of discussions about health-related information in society, such as the use and effects of drugs, personal experiences, price evaluations, and adverse reactions^22^. Given this background, we analyzed NAIs associated with age-related AEs using the U.S. FDA AE reporting systems (FAERS) and the WebMD internet message boards.

## Results

### Characteristics of the study population from the FAERS

Each case patient has more than one preferred term for a specific drug reported, therefore resulting in more than one drug-AE pair for each AE report. In total, 16729 AEs from 4598 patients from January of 2013 to December of 2018 were included in the analysis of the NAIs and AEs from the FAERS data. The characteristics of the patients and the AE reports from the FAERS are presented in Table 1. The mean age of the patients was 30.54 and 58.75 years for oseltamivir and peramivir, respectively. A mean of 3.83 for AEs on average was reported per patient administered with oseltamivir, and these patients were co-administered with a mean of 8.21 for other drugs. AEs were more common among younger patients (73 out of 238, 30.67%) for zanamivir, while they were more common for older patients (94 out of 148 AEs, 63.51%) for peramivir.

**Table 1:** Demographic characteristics of the subjects from the FAERS data.

Characteristics	Oseltamivir	Laninamivir	Peramivir	Zanamivir
**Number** **of** **patients,** *n*	4200	12	148	238
**Age, years**	30.54 ± 30.44	53.57 ± 16.89	58.75 ± 30.22	28.63 ± 24.92
Less than 19 years old, *n* (%)	771 (18.36)	10 (83.33)	15 (10.14)	73 (30.67)
19 to 64 years old, *n* (%)	1414 (33.67)	1 (8.33)	34 (22.97)	94 (39.50)
More than 64 years old, *n* (%)	817 (19.45)	1 (8.33)	94 (63.51)	28 (11.76)
Unknown, *n* (%)	1198 (28.52)	0	5 (3.38)	43 (18.07)
**Gender (*n*** **males, %)**				
Male, *n* (%)	1623 (38.64)	6 (50.00)	67 (45.27)	100(42.02)
Female, *n* (%)	2206 (52.52)	6 (50.00)	71 (47.97)	108 (45.38)
Unknown, *n* (%)	371 (8.83)	0	10 (6.76)	30 (12.61)
**Occurrence country, n (%)**				
North America, Europe, Oceania	2994 (71.29)	0	0	70 (29.41)
South America	130 (3.10)	0	0	0
Asia	553 (13.17)	12 (100.00)	101 (68.24)	133 (55.88)
Unknown	523 (12.45)	0	47 (31.76)	35 (14.71)
**Duration of administration (days)**	3.06 ± 7.71	3.17 ± 2.03	1.28 ± 1.42	2.81 ± 2.45
**Coadministration drugs**, *n*	8.21 ± 11.49	19.75 ± 17.27	4.62 ± 7.57	5.36 ± 8.65
**Reported events per patients**, *n*	3.83 ± 2.89	8.40 ± 5.84	2.40 ± 1.74	3.47 ± 2.28

Values are reported as n (%) or mean ± standard deviation. FAERS, Food and Drug Administration Adverse Event Reporting System.

### AEs by NAIs from the FAERS

The system organ classes of AEs reported for NAIs are shown in Table 2. AEs were most frequently reported for oseltamivir (15409, 92.11%), followed by zanamivir (891, 5.33%), peramivir (345, 2.60%), and laninamivir (84, 0.50%). Psychiatric disorders (1880, 12.20%) were the most common AE clinical symptoms for oseltamivir. The occurrence rates of psychiatric disorders of zanamivir, laninamivir, and peramivir were 9.09%, 2.38%, and 1.16%, respectively. Cardiac and vascular disorders (9.57% and 9.28%, respectively) were the most common AEs for peramivir, while general disorders and administration site conditions (113, 12.68%) were the most common AEs for zanamivir. A disproportionality analysis showed that the signals of vomiting, hallucination, headache, insomnia, fatigue, and dizziness were detected for oseltamivir (Table 3). There was no AE signal defected for laninamivir, peramivir, and zanamivir. Therefore, a further subgroup analysis was conducted with younger and elderly patients. The disproportionality analysis showed that signals for vomiting and hallucinations were detected in younger patients (<19 years) given oseltamivir, while an abnormal hepatic function, cardiac failure, shock and cardio-respiratory arrest were detected in older patients (>65 years) given peramivir (Table 4).

**Table 2:** Adverse events associated with NAIs in subjects from the FAERS data.

SOC terms	Oseltamivir, *n* (%) (*N* = 15409)	Laninamivir, *n* (%) (*N* = 84)	Peramivir, *n* (%) (*N* = 345)	Zanamivir, *n* (%) (*N* = 891)
Psychiatric disorders	1880 (12.20)	2 (2.38)	4 (1.16)	81 (9.09)
Gastrointestinal disorders	1786 (11.59)	18 (21.43)	22 (6.38)	56 (6.29)
General disorders and administration site conditions	1875 (12.17)	7 (8.33)	32 (9.28)	113 (12.68)
Respiratory, thoracic and mediastinal disorders	1355 (8.79)	2 (2.38)	23 (6.67)	99 (11.11)
Nervous system disorders	1169 (7.59)	6 (7.14)	25 (7.25)	99 (11.11)
Skin and subcutaneous tissue disorders	672 (4.36)	2 (2.38)	12 (3.48)	32 (3.59)
Musculoskeletal and connective tissue disorders	577 (3.74)	4 (4.76)	7 (2.03)	8 (0.90)
Cardiac disorders	609 (3.95)	0	33 (9.57)	35 (3.93)
Vascular disorders	448 (2.91)	4 (4.76)	32 (9.28)	41 (4.60)
Others^a^	5038 (32.70)	39 (46.43)	155 (44.93)	327 (36.70)

FAERS, Food and Drug Administration Adverse Event Reporting System; SOC, System Organ Classes; ^a^Blood and lymphatic system disorders, Immune system disorders, Infections and infestations, Injury, poisoning and procedural complications, Metabolism and nutrition disorders, Renal and urinary disorders, etc.

**Table 3:** Signal detection of clinical symptoms reported for adverse events associated with a neuraminidase inhibitor, oseltamivir.

Clinical symptoms	Event (n)	PRR (kai^2^)	ROR (95% CI)	IC
Vomiting	379	3.25 (15.51)	3.30 (1.76, 6.21)	0.08
Hallucination	209	4.48 (10.73)	4.52 (1.68, 12.19)	0.09
Headache	145	4.14 (7.06)	4.17 (1.33, 13.10)	0.09
Insomnia	128	3.66 (5.70)	3.68 (1.17, 11.57)	0.09
Fatigue	114	9.77 (7.85)	9.83 (1.37, 70.45)	0.11
Dizziness	112	9.59 (7.68)	9.66 (1.35, 69.22)	0.11

PRR, proportional reporting ratio; ROR, reporting odds ratios; CI, the confidence interval.

**Table 4:** Signal detection for adverse events associated with neuraminidase inhibitors in vulnerable subjects.

Clinical symptoms	Event (n)	PRR (kai^2^)	ROR (95% CI)	IC
**Oseltamivir <19 years**				
Vomiting	118	8.51 (13.68)	8.3 (2.20, 36.30)	0.19
Hallucination	103	7.43 (11.38)	7.74 (1.90, 31.52)	0.19
**Peramivir ≥65 years**				
Hepatic function abnormal	9	26.08 (77.83)	27.17 (9.02, 81.79)	3.36
Cardiac failure	5	24.14 (41.70)	24.69 (5.86, 104.03)	3.36
Cardio-respiratory arrest	5	18.11 (36.01)	18.51 (4.93, 69.45)	3.21
Shock	5	12.07 (27.79)	12.33 (3.73, 40.75)	2.94

PRR, proportional reporting ratio; ROR, reporting odds ratios; CI, the confidence interval.

### Characteristics of the study population from the WebMD

In total, 396 review comments and 440 subjects from 413 reviewers from Oct 2007 to May 2019 were included in the NAI-associated AEs analysis after excluding instances with no comments from the WebMD data. The characteristics of the subjects from WebMD are presented in Table 5. These subjects consisted of 74 younger (16.82%), 318 adult (72.27%), and 25 older subjects (5.68%). Review comments were most frequently reported for oseltamivir at 418 (95.00%), followed by zanamivir at 21 (4.77%). The content themes of the review comments contained mostly reasons why the medicines were being taken (270, 35.52%) and AEs (288, 37.89%).

**Table 5:** Demographic characteristics of the subjects from the WebMD data.

Characteristics	Values
**Number of subjects**, *n*	440
**Age, years**	
Less than 19 years old, *n* (%)	74 (16.82)
19 to 64 years old, *n* (%)	318 (72.27)
More than 64 years old, *n* (%) elderly	25 (5.68)
Unknown, *n* (%)	23 (5.23)
**Gender (*n*** **males, %)**	
Male, *n* (%)	110 (25.00)
Female, *n* (%)	269 (61.14)
Unknown, *n* (%)	61 (13.86)
**Medicines**	
Oseltamivir	418 (95.00)
Zanamivir	21 (4.77)
Peramivir	1 (0.23)
**Theme**^a^	724
Why to take the drug	270 (35.52)
Effectiveness after taking	118 (15.53)
Adverse events	288 (37.89)
Price	48 (6.32)

^a^Total number of themes counted in each review. This is greater than the total number of reviewers because some reviews had more than one theme tied for the concern.

### AEs by NAIs from the WebMD

AEs were most frequently reported for oseltamivir (525, 96.33%), followed by zanamivir (20, 3.67%). Among those taking oseltamivir, psychiatric disorders (162, 30.86%) were the most common symptoms, followed by gastrointestinal disorders (157, 29.90%) and cardiac disorders (46, 8.76%) (Table 6). Psychiatric disorders were most common in younger (7.56%) and older (3.92%) patients, while gastrointestinal disorders were most common in adult patients (35.85%) given oseltamivir (Table 7).

**Table 6:** Frequencies of adverse events associated with NAIs from patient reviews in the WebMD.

SOC terms	Oseltamivir, *n* (%) (*N* = 525)	Zanamivir, *n* (%) (*N* = 20)
Psychiatric disorders	162 (30.86)	0
Gastrointestinal disorders	157 (29.90)	10 (50.00)
Cardiac disorders	46 (8.76)	2 (10.00)
Nervous system disorders	41 (7.81)	3 (15.00)
Skin and subcutaneous tissue disorders	32 (6.10)	0
General disorders and administration site conditions	18 (3.43)	1 (5.00)
Respiratory, thoracic and mediastinal disorders	13 (2.48)	0
Musculoskeletal and connective tissue disorders	9 (1.71)	0
Others^a^	47 (8.95)	4 (20.00)

SOC, System Organ Classes; ^a^Ear and labyrinth disorders, Immune system disorders, Infections and infestations, Injury, poisoning and procedural complications, Metabolism and nutrition disorders, Product issues, Renal and urinary disorders, Reproductive system and breast disorders, Vascular disorders.

**Table 7:** Frequencies of adverse events associated with NAIs from patient reviews according to their ages in the WebMD data.

Age	SOC terms	Oseltamivir, *n* (%) (N = 357)
<19 years	Psychiatric disorders	27 (7.56)
	Gastrointestinal disorders	21 (5.88)
	Cardiac disorders	6 (1.68)
19–64 years	Psychiatric disorders	118 (33.05)
	Gastrointestinal disorders	128 (35.85)
	Cardiac disorders	35 (9.80)
≥65 years	Psychiatric disorders	14 (3.92)
	Gastrointestinal disorders	4 (1.12)
	Cardiac disorders	4 (1.12)

SOC, System Organ Classes.

## Discussion

NAIs remain a widely licensed class of antiviral drugs appropriate for the treatment and prophylaxis of seasonal influenza^23^. However, there is still concern regarding the adverse effects of NAIs. This study analyzed the age-related AEs associated with NAIs using data from FAERS and WebMD.

The results of this study demonstrated that the occurrence rate of AEs and adverse symptoms varied and depended on the NAIs used and the age of the patient, despite the considerable degree of structural similarity. Oseltamivir was the NAI most commonly showing AEs in the FAERS data, and the most common AEs for this drug were psychiatric and gastrointestinal disorders, similar to the findings of previous studies^8,13–16^. For zanamivir, the most common AEs were general disorders and administration site conditions, consistent with a previous report^4^. The signal detection PRR, ROR, and IC methods were able to detect several AEs associated with oseltamivir only in the FAERS data. The most likely cause is the extremely low number of AE cases for other NAIs. To support our results, sensitivity analyses were conducted using the disproportionality method stratified according to gender or type of reporter. Similar trends were observed in other sensitivity analysis that limited the data further via certain gender or health professional reporters. Additionally, AE signals for vomiting and hallucinations were detected in younger patients given oseltamivir, while an abnormal hepatic function, cardiac failure, shock and cardio-respiratory arrest were detected in older patients given peramivir. However, in the WebMD data, we could not detect signals by these disproportionality analyses due to the small number of AE cases, although psychiatric and gastrointestinal disorders were the most common AEs reported. The numbers of the younger and older subjects were quite low compared to the number of adults in the WebMD data, possibly due to the low rate of accessibility to the internet or digital devices and/or the recognition of the need to report.

Oseltamivir phosphate is an oral prodrug which undergoes hydrolysis by hepatic esterases to convert an active metabolite, oseltamivir carboxylate^24^. Oseltamivir can induce neuropsychiatric adverse effects with either a sudden or delayed onset. Sudden-onset reactions are due to the direct effects of oseltamivir on the central nervous system, whereas delayed-onset reactions are due to the effects of oseltamivir carboxylate^24–26^. Oseltamivir phosphate itself can cause the central depressant actions that may result in abnormal behavior, delirium, hallucinations, sleep, and respiratory depression^26^. Oseltamivir phosphate can inhibits nicotinic acetylcholine receptors and monoamine oxidase A. Additionally, gamma-aminobutyric acid receptors and N-methyl-D-aspartate and their related receptors/channels are thought to be other candidates related to respiratory suppression^24^. It has been shown that while oseltamivir carboxylate cannot pass through the blood-brain barrier (BBB) readily, it may do so when combined with other agents or when the BBB is immature or impaired^12^. Most oseltamivir-induced neuropsychiatric adverse effects have been reported in Asian populations^14,15,26^. Li *et al*. hypothesized that a nonsynonymous single-nucleotide polymorphism rs2233385, near the active site of human cytosolic sialidase which is a homolog of the virus neuraminidase and presents in Asian populations (9.29%), could increase the binding affinity of sialidase to oseltamivir carboxylate, thus reducing the sialidase activity and contributing to the occurrence of severe neuropsychiatric adverse effects^27^.

It has been reported that the most commonly reported events when using zanamivir as a treatment were gastrointestinal and respiratory, thoracic and mediastinal disorders, but the incidences were similar to those in a placebo group^28^. The most frequently reported AEs of laninamivir were gastrointestinal disorders (27.0%), psychiatric disorders (26.8%), and skin disorders (18.7%) during early post-marketing phase vigilance^29^. The AEs of peramivir were a high mortality rate, the development of acute respiratory distress syndrome (7%), and renal failure (5%) in the FAERS system^30^. These results were similar to the results here. However, an abnormal hepatic function and cardiac failure were detected in older patients given peramivir in our results. To the best our knowledge, this is the first study that compares NAI-related AEs in senior citizen patients using FAERS data. Because it is noteworthy that cardiovascular risks are high in older persons, care must be taken when administering these drugs in this population.

A disproportionality analysis alone is not sufficient proof of the drug AE association nor a causality assessment of an individual report^31^. There is no way to determine exactly how many people took a particular drug, nor is there any method by which to ascertain how many events occurred when they took the drug. Therefore, in our study, we do not know actual real number of patients exposed to NAIs. Accordingly, it is difficult to assess the relationship between their AEs and incidence rates. It has been reported that there were 2, 102, 885 prescriptions of oseltamivir capsules, 494, 188 prescriptions of oseltamivir powder, and 7, 955 prescriptions of zanamivir among 101, 947, 808 outpatients from 2010 to 2015 in the Sentinel System of the Centers for Disease Control and Prevention’s Influenza-like Illness Surveillance Network System and National Respiratory and Enteric Virus Surveillance System^32^.

Pharmacoepidemiological methods as presented in case-control or cohort studies are considered as the best sources of drug safety data. However, they are also associated with several unfavorable methodological issues, such as a limited sample size, reduced follow-up, and evaluations of surrogate markers^33^. Fortunately, a quantitative means of comparing the AE rates of one drug with those of all other drugs is now available. Nevertheless, the outcomes of comparisons of AE rates through a disproportionality analysis can be influenced by many factors, including absolute report numbers, the presence of other AEs associated with the same drug, significant heterogeneity, and potential bias issues such as physician preference for one drug over others and a patient’s negative experience with a certain product along with reporting biases such as underreporting^34^. Additionally, other factors that can influence the association between the frequency of AE reporting and medicine include the length of time the medicine has been marketed and the extent of publicity about new safety concerns. Furthermore, with this approach it is necessary for each AE report to be validated before any analysis in the context of the pharmacological and medical hypothesis of the study in order to prevent false results^35^.

Another limitation of this study was that the reported numbers of adverse cases for NAIs were low, except for that associated with oseltamivir, possibly because the AE reports were submitted voluntarily, leading to their being underreported, or there may have been cases which lacked approval by the U.S. FDA. However, the early detection of AEs and risk evaluations in vulnerable populations are important. Continued efforts with regard to the identification and evaluation of AEs associated with NAIs as well as to understand their underlying susceptibility mechanisms, are needed to treat and manage AEs more efficiently in patients with influenza.

In conclusion, AEs associated with NAIs were analyzed using data from the two databases. Serious AEs associated with NAIs may have a significant impact on younger or older patients. From the findings here, younger or older patients should be monitored carefully for AEs when treated with NAIs.

## Methods

### Data collection

The study population consisted of patients reported to have AEs in the FAERS and WebMD datasets. Reported AE cases related to NAIs from the FAERS database were used in this study. AEs and medication errors are coded to terms in the Medical Dictionary for Regulatory Activities (MedDRA) terminology^36^. The FAERS data from 2013 to 2018 were downloaded. Duplicated reports were deleted according to the U.S. FDA’s recommendation of adopting the most recent case number. The drug lexicon was devised using both the generic and trade names in the FAERS database. The available NAIs selected for the analysis were oseltamivir, peramivir, zanamivir, and laninamivir. The preferred term and the system organ classes in the MedDRA were used for further analysis. Two or more preferred terms reported in one patient were counted as different AEs. Instances of co-administration with NAIs were excluded from any further analysis. For text mining, subject comments pertaining to oseltamivir, peramivir, and zanamivir downloaded from WebMD^37^ using the python-based library Beautiful Soup^38^. The WebMD data from Oct 2007 to May 2019 were downloaded. Each reported symptom was assigned the preferred terms in the MedDRA terminology manually. Information about age, sex, condition, reviewer types, report date, and treatment duration was collected.

### Statistical analysis

Patients were classified into three age groups: children and adolescents (age <19 years), adults (age 19–64 years) and older people (age ≥65 years). The MedDRA preferred term was used for a quantitative disproportionality analysis. Disproportionality was analyzed using the proportional reporting ratio (PRR)^39^, the reporting odds ratio (ROR)^40^, and the information component (IC) methods^41^. The PRR is calculated according to the ratio of the proportion of all reported cases of the event of interest among people exposed to a particular drug to the corresponding proportion among people exposed to all or several other drugs^39^. The ROR is calculated according to ratio of the odds of the reporting of one specific event versus all other events for a given drug relative to the matching reporting odds for all other drugs^40^. The IC measure can be considered as the calculation of the logarithm of the ratio of the observed rate of reporting of a specific drug-AE combination to the expected rate under the null hypothesis of no association between the drug and AE^41^. For the PRR, a given drug AE pair was defined as a signal if the event count was 3 or more, while the PRR was 2 or more with an associated chi-square value of 4 or more^39^. For the ROR, it was defined if the lower limit of the 95% two-sided confidence interval (CI) of ROR exceeded 1^40^. The information component (IC) algorithm performs signal detection via the IC025 metric, which is a lower bound of the 95% two-sided confidence interval of IC, with an AE signal indicated when the IC025 value exceeds 0^41^. For the WebMD data, the frequency of each theme of a patient medication concern was computed for each comment, with these outcomes then summed for all comments. Data were analyzed with Microsoft EXCEL 2016 (Microsoft, Redmond, WA, USA) and SAS version 9.4 (SAS Institute Inc., Cary, NC, USA).

## Data Availability

The datasets generated during the current study are available from the corresponding authors on reasonable request.
